# 24R,25-Dihydroxyvitamin D3 Protects against Articular Cartilage Damage following Anterior Cruciate Ligament Transection in Male Rats

**DOI:** 10.1371/journal.pone.0161782

**Published:** 2016-08-30

**Authors:** Barbara D. Boyan, Sharon L. Hyzy, Qingfen Pan, Kayla M. Scott, Richard D. Coutts, Robert Healey, Zvi Schwartz

**Affiliations:** 1 Department of Biomedical Engineering, School of Engineering, Virginia Commonwealth University, Richmond, Virginia, United States of America; 2 Wallace H. Coulter Department of Biomedical Engineering at Georgia Tech and Emory University, College of Engineering, Georgia Institute of Technology, Atlanta, Georgia, United States of America; 3 School of Mechanical Engineering, College of Engineering, Georgia Institute of Technology, Atlanta, Georgia, United States of America; 4 Department of Orthopedic Surgery, University of California San Diego, San Diego, California, United States of America; 5 Department of Periodontics, University of Texas Health Science Center at San Antonio, San Antonio, Texas, United States of America; Ohio State University, UNITED STATES

## Abstract

Osteoarthritis (OA) in humans is associated with low circulating 25-hydroxyvitamin D3 [25(OH)D_3_]. In vitamin D replete rats, radiolabeled 24R,25-dihydroxyvitamin D3 [24R,25(OH)_2_D_3_] accumulates in articular cartilage following injection of [^3^H]-25(OH)D_3_. Previously, we showed that 24R,25(OH)_2_D_3_ blocks chondrocyte apoptosis via phospholipase D and p53, suggesting a role for 24R,25(OH)_2_D_3_ in maintaining cartilage health. We examined the ability of 24R,25(OH)_2_D_3_ to prevent degenerative changes in articular cartilage in an OA-like environment and the potential mechanisms involved. In vitro, rat articular chondrocytes were treated with IL-1β with and without 24R,25(OH)_2_D_3_ or 1α,25(OH)_2_D_3_. 24R,25(OH)_2_D_3_ but not 1α,25(OH)_2_D_3_ blocked the effects of IL-1β in a dose-dependent manner, and its effect was partially mediated through the TGF-β1 signaling pathway. In vivo, unilateral anterior cruciate ligament transections were performed in immunocompetent rats followed by intra-articular injections of 24R,25(OH)_2_D_3_ or vehicle (t = 0, 7, 14, 21 days). Tissues were harvested on day 28. Joints treated with vehicle had changes typical of OA whereas joints treated with 24R,25(OH)_2_D_3_ had less articular cartilage damage and levels of inflammatory mediators. These results indicate that 24R,25(OH)_2_D_3_ protects against OA, and suggest that it may be a therapeutic approach for preventing trauma-induced osteoarthritis.

## Introduction

Osteoarthritis (OA) is a degenerative condition that affects 12.1% of the US population over the age of 25 [1] and is the leading cause of disability in the elderly [2]. It is characterized by fibrillation and eventual erosion of the articular cartilage, ultimately exposing the subchondral bone. More recently, it has become evident that OA involves all of the tissues of the affected joint [3,4], but there is not yet agreement what role each tissue plays in the overall condition and on the mechanisms involved. During the development of OA, the bone underlying the articular cartilage becomes hypermineralized and stiffer [5], altering the mechanical properties of the joint and the transport of nutrients into the cartilage tissue from the bone vasculature and marrow [6]. The synovial fluid within the joint space exhibits altered chemistry, including increased content of inflammatory cytokines and products of cartilage matrix breakdown [7]. The synovium, which is an innervated tissue, also shows changes [8], suggesting that it is responsible for the pain associated with OA in addition to being a source of inflammatory cytokines.

The complex cellular and extracellular matrix (ECM) architecture of the articular cartilage is markedly affected as OA develops. The ability of the cells that line the articulating surface to produce lubricin is lost [9], resulting in altered lubrication during mechanical loading [10]. As the integrity of the ECM is reduced via the action of matrix metalloproteases and other matrix processing enzymes, the diffusivity properties are altered. Clonal populations of chondrocytes in the mid layer of the cartilage begin to differentiate and to produce mineralized ECM vesicles [11], further modifying the mechanical properties of the tissue. To better understand the events at a cell level, investigators have characterized the regulatory mechanisms involved in matrix degradation, particularly the roles of inflammatory cytokines like interleukin-1 beta (IL-1β) in modulating expression, synthesis, and activity of acid matrix metalloproteinases (MMPs) [12–14].

Treatment of OA is primarily palliative until it becomes sufficiently painful to warrant total joint replacement. The factors that influence the rate and extent of OA progression include mechanical stability of the joint and hormonal regulation [15,16]. Trauma to the joint, particularly trauma to the anterior cruciate ligament (ACL), is a major risk factor for OA [17,18]. For this reason, in vivo studies examining OA development or that test potential pharmaceutical interventions frequently use rats in which the ACL is transected, leading to joint instability [19–21]. Many of the drugs tested using the anterior cruciate ligament transection (ACLT) model address the problem of inflammation using a protocol designed to reverse the damage due to the mechanical insult. We approached the problem from the hypothesis that the damage can be prevented by blocking chondrocyte apoptosis and matrix degradation due to inflammatory stimuli.

A number of studies in our lab and others led us to consider the vitamin D metabolite 24R,25-dihydroxyvitamin D_3_ [24R,25(OH)_2_D_3_] as a candidate to achieve this goal. 24R,25(OH)_2_D_3_ stimulates proliferation and ECM synthesis of chondrocytes from the costochondral cartilage reserve zone, a hyaline-like cartilage tissue [22,23]. The mechanism is phospholipase D (PLD) dependent and involves up-regulation of p53. 24R,25(OH)_2_D_3_ also blocks apoptosis in chondrocytes caused by several stimuli [24–26]. Its importance to cartilage health was suggested by the observations that vitamin D replete rats concentrate radiolabeled 24R,25(OH)_2_D_3_ in the articular cartilage when injected with tritiated 25-hydroxyvitamin D_3_ [25(OH)D_3_] [27] and that chondrocytes can synthesize 24,25(OH)_2_D_3_ under regulation by transforming growth factor beta-1 (TGF-β1) [28], a known chondrogenic growth factor [29]. Also, 24R,25(OH)_2_D_3_ stimulates the production of latent transforming growth factor binding protein [30], thereby regulating the storage of latent TGF-β1 in the ECM and suggesting that the two factors may function in a cooperative manner to preserve cartilage health. In contrast, the other well-known active vitamin D3 metabolite 1α,25(OH)_2_D_3_ has been shown to increase inflammatory processes [31] and cartilage erosion [32] in articular cartilage, making it a poor candidate for a possible therapeutic.

Clinical evidence has associated OA with reduced serum 25(OH)D_3_ [33], raising the possibility that 25(OH)D_3_ would also be reduced in OA synovial fluid, which was confirmed in synovial fluid harvested from humans with advanced OA [34]. Reduced 25(OH)D_3_ limits further metabolism to 24R,25(OH)_2_D_3_ [35]. This suggested to us that by increasing the synovial fluid concentration of 24R,25(OH)_2_D_3_, we would mitigate negative effects due to ACL trauma *in vivo*. Therefore, the aim of our study was to assess the chondroprotective effects of 24R,25(OH)_2_D_3_ in a chondrocyte cell culture model and whether it mitigates cartilage changes and inflammation in an *in vivo* ACL transection model of osteoarthritis.

## Materials and Methods

### Reagents

Rat IL-1β was purchased from PeproTech (Rocky Hill, NJ). 24R,25(OH)_2_D_3_ and 1α,25(OH)_2_D_3_ were obtained from Enzo Lifesciences (Farmingdale, NY). TGF-β1 was purchased from R&D Systems (Minneapolis, MN). All other reagents were purchased from Sigma-Aldrich (St. Louis, MO) unless specified.

### Cell culture and articular chondrocyte phenotype characterization

Articular cartilage was obtained from the femurs of 100–125 gram male Sprague Dawley rats under Virginia Commonwealth University Institutional Animal Care and Use Committee (IACUC) approved protocol AD10000642. Animals were euthanized by CO2 asphyxiation using a pre-calibrated 3.5 L/min flow rate designed to minimize distress. Cartilage specimens were thinly sliced and incubated in 0.25% trypsin for 30 min at 37°C. Rat articular chondrocytes were extracted by incubating the cartilage fragments for 16 hours in Dulbecco’s modified Eagle’s medium (DMEM, Thermo Fisher, Waltham, MA), 1% penicillin-streptomycin (Life Technologies, Carlsbad, CA), and 0.03% collagenase type II (Worthington Biosciences, Lakewood, NJ). This solution was passed through 40μm cell strainers to remove tissue debris. Cells were cultured in 75 cm^2^ flasks in DMEM supplemented with 10% fetal bovine serum (Life Technologies) and 1% penicillin–streptomycin at 37°C in a humidified atmosphere containing 5% CO_2_. Cells were expanded in culture until passage 4. At the end of each passage, RNA was extracted, and expression of chondrocyte genes determined using real-time PCR. First-passage cells were chosen for experiments based on their high levels of expression of chondrocyte markers. First-passage cells were plated for experiments at a density of 15,000 cells/cm^2^, and media were changed 24 hours after plating and every 48 hours thereafter until cells reached confluence.

### *In vitro* experimental design

Rat IL-1β was reconstituted in sterile PBS containing 0.1% bovine serum albumin to a stock concentration of 100μg/ml. The stock was diluted to final concentrations in culture medium. Confluent cell cultures were treated with 1, 5, or 10ng/ml IL-1β for 24 hours to determine the dose for later experiments. Caspase-3 activity and DNA fragmentation were determined using cell lysates as described previously [36]. NO production in the conditioned media was determined using a 2,3-diaminonaphthlene assay (DAN) [37]. Prostaglandin E2 (PGE_2_) and matrix metalloproteinase-13 (MMP-13) activity were measured in the conditioned media using kits following the manufacturer’s directions.

To examine the effect of 24R,25(OH)_2_D_3_ on IL-1β stimulated chondrocytes, confluent cultures of rat articular chondrocytes were treated with 10 ng/ml IL-1β for 12 hours. For the next 12 hours, cells were treated with 10 ng/ml of IL-1β or 10 ng/ml of IL-1β with 10^−9^–10^−7^ M of 24R,25(OH)_2_D_3_. Chondrocyte gene expression was determined using real-time PCR and levels of NO, PGE_2_, and MMP-13 were measured as described above. To assess the specificity of the effect of 24R,25(OH)_2_D_3_, confluent cultures of rat articular chondrocytes were treated for 12 hours with 10 ng/ml IL-1β. For the next 12 hours, cells were treated with 10 ng/ml IL-1β or 10 ng/ml IL-1β with 10^−7^ M 24R,25(OH)_2_D_3_ or 10^−8^ M 1α,25(OH)_2_D_3_. The dose used for each metabolite was based on prior studies [26,38]. MMP-13 activity was used as the outcome measure.

To examine the protective effect of TGF-β1 on chondrocytes stimulated by IL-1β, confluent cultures were treated with 10 ng/ml IL-1β for 12 hours. For the next 12 hours, cells were treated with 10 ng/ml IL-1β or 10 ng/ml IL-1β with 0.1, 1, or 10 ng/ml TGF-β1. Levels of NO, PGE_2_, and MMP-13 were measured.

To investigate the interaction of 24R,25(OH)_2_D_3_ and TGF-β1 on MMP-13 activity, confluent rat chondrocytes were treated with 10^−8^ M 24R,25(OH)_2_D_3_ ± 0.1 ng/ml TGF-β1 for the last 12 hours in addition to the 10 ng/ml IL-1β treatment.

24R,25(OH)_2_D_3_’s effect on regulating TGF-β1 production and signaling was examined by treating confluent rat articular chondrocytes with 10^−9^–10^−7^ M 24R,25(OH)_2_D_3_. TGF-β1 was measured in the conditioned media via ELISA (R&D Systems) [39]. Total TGF-β1 was measured by acidifying the conditioned media in HCl for 10 min followed by neutralization with NaOH. Active TGF-β1 was measured in unacidified conditioned media. Latent TGF-β1 levels were calculated by subtracting the active TGF-β1from the total TGF-β1. Secreted TGF-β1 was normalized to DNA content of the cell lysates. mRNA expression of TGF-β1 receptor type II (Tgfbr2), and signaling molecules Smad2 and Smad3 was measured using real-time PCR as described below.

To examine if the effect of 24R,25(OH)_2_D_3_ on MMP-13 activity was mediated by TGF-β1, confluent rat articular chondrocytes were treated with IL-1β for 12 hours and prior to the addition of combination of IL-1β and 24R,25(OH)_2_D_3_, 2 μg/ml antibody for blocking the type II TGF-β1 receptor (R&D Systems, AF-241), or a soluble ligand binding receptor peptide (R&D Systems) was added to the media for 30 min. Outcomes were measured at 12 hours using ELISA assays (R&D Systems). The goat polyclonal anti-human type II TGF-β1 receptor antibody was raised against Ile24-Asp159 (Human), which has 79.9% homology to rat P38438 [88.9% positive amino acid recognition].

#### Gene expression

RNA was harvested using a TRIzol® (Life Technologies) extraction method following the manufacturer’s protocol. mRNA was quantified using a NanoDrop spectrophotometer (Thermo Fisher Scientific, Waltham, MA). RNA (250 ng) was amplified using reverse transcription (High Capacity cDNA Reverse Transcription Kit, Life Technologies). Starting quantities of mRNA were determined using SybrGreen chemistry (Power SYBR® Green PCR Master Mix, Life Technologies) in a StepOne Plus imaging system (Life Technologies) using gene-specific primers (Table 1). mRNA levels for chondrocyte genes encoding aggrecan (Acan), type II collagen (Col2), and sex determining region Y-box 9 (Sox9), as well as for Smad2, Smad3, and Tgfbr2 were measured by quantitative real-time PCR (qPCR). All mRNAs are presented as normalized to glyceraldehyde-3-phosphate dehydrogenase (GAPDH).

**Table 1:** Primer sequences used in real-time qPCR analyses.

Gene Name	Accession Number	Forward Primer	Reverse Primer
Acan	NM_022190.1	GCTTCGCTGTCCTCAATGC	AGGTGTCACTTCCCAACTATCC
Col2a1	NM_012929.1	GCTTCTTCTCCTTGCTCTTGC	TGGCGAGTCTTGCGTCTAC
Sox9	XM_001081628.5	ATCGGAGCGGAGGAGGAG	GTGGGAGCGACAACTTTACC
Comp	NM_012834.1	TCCCCGTCCTGGTCTTGG	AGTGACAGCGATGGTGATGG
Col10a1	XM_008773017.1	ATAGTGCTGCTGCCTGTTG	TTTCTGGGATGCCTCTTGTC
Col1a1	NM_053304.1	AGTGATAGGTGATGTTCTGG	CGAGTATGGAAGCGAAGG
Smad2	NM_019191.1	TCCTGTCCATTCTGTTCTCC	CGTCCATCTTGCCATTCAC
Smad3	NM_013095.3	CGACCACCAGATGAACCACA	CGACCACCAGATGAACCACA
Tgfbr2	NM_031132	ACAGCGTTGCAGCGCGACGT	ATGACCTGGCCAACAGCGGGCA
Gapdh	NM_017008	CATACTCAGCACCAGCATCACC	AAGTTCAACGGCACAGTCAAGG

#### DNA fragmentation

At 90% confluence, cells were incubated with 1 μCi/ml ^3^H-thymidine (Perkin Elmer, Waltham, MA) for four hours before IL-1β treatment. Cells were then treated for 24 hours with 10 ng/ml IL-1β. At the end of the treatment period, cell monolayers were lysed [10mM Tris-HCl, 1mM EDTA, 0.2% Triton X-100] and then were subjected to three freeze-thaw cycles. Intact DNA was separated from fragmented DNA by ultracentrifugation at 13,000*g* for 15 min. Intact DNA (pellet) and fragmented DNA (supernatant) were measured by liquid scintillation counting. Results are presented as percent fragmented DNA/total DNA.

#### Caspase-3 activity

Caspase-3 activity was determined using a colorimetric assay (CaspACE® Assay, Promega, Madison, WI). Monolayers were lysed in cold lysis buffer for 10 min at 4°C, and the cell lysates centrifuged at 10,000g for 1 minute. The resulting supernatant was combined with 2x reaction buffer and DEVD-pNA substrate and incubated at 37°C for 2 hours. Absorbance at 405 nm was determined using a microplate reader (VersaMax, Molecular Devices, Sunnyvale, CA). Caspase-3 activity was normalized to total protein content (Pierce 660nm Protein Assay, Thermo Fisher Scientific).

#### Nitric oxide

Nitric oxide in the conditioned media was measured using a 2,3-diaminonaphthalene (DAN) fluorescent assay by measuring the total amount of nitrite and nitrate in the media. NO production was normalized to total DNA (Promega, Madison, WI).

#### Prostaglandin E2

PGE_2_ production was measured in conditioned media using a competitive enzyme immunoassay (R&D Systems, Minneapolis, MN), and normalized to total DNA (Promega).

#### Matrix metalloproteinase-13 activity

MMP-13 activity in conditioned media was determined using a fluorometric assay kit (AnaSpec, Fremont, CA). To determine MMP-13 activity, a monoclonal anti-human-anti-MMP13 was used to pull down both pro- and active forms of MMP-13. The activity of MMP-13 was quantified by a 5-FAM/OXL 520 fluorescence resonance energy transfer (FRET) peptide and normalized to total DNA (Promega).

#### Statistical analysis

Data are presented as mean ± SEM of n = 6 independent cultures per variable. All experiments were repeated to validate the results. Data presented are from one representative experiment of two trials. Data were examined by analysis of variance (ANOVA) and post hoc test using Bonferroni’s modification of Student’s t-test. P<0.05 was considered to be significant. The treatment/control value was calculated by dividing the value of each sample from the treated group by the mean of the control group. Each data point represents the mean ± SEM for six normalized values; the control value of 1 is indicated using a dashed line. Significance was determined by Mann-Whitney test. p ≤ 0.05 was considered to be significant.

### ACL transection model study design

The anterior cruciate ligament transection model was selected for this study based on studies showing that the damage to the articulating surface of the joint is similar to what is seen in post-traumatic osteoarthritis [40]. We hypothesized that the anti-apoptotic effects of 24R,25(OH)_2_D_3_ observed on growth plate chondrocytes in vitro [25,26] would mitigate tissue damage due to inflammation associated with transection of the ACL and disruption of the normal mechanical properties of the joint. To test this hypothesis, the vitamin D metabolite was administered by injection into the joint space immediately after ACL transection and then at seven-day intervals (S1 Fig). The study was terminated at 28 days based on literature indicating that tissue damage would be evident at that time point in the absence of treatment [20,40]. A power analysis was used to determine the number of animals in each of the two cohorts (10 rats per group: ACL transection followed by vehicle only injections v. ACL transection treated with 24R,25(OH)_2_D_3_). All animals received injections in the right knee, and the left knee was used as an internal control for changes due to altered weight bearing. Rats were randomized into each cohort. No rats were lost during the study, either as a result of surgery or treatment.

The 25 μL injection volume was selected so that the test articles would be diluted by four in the ~100uL of synovial fluid in the joint. 4*10^-7^M 24R,25(OH)_2_D_3_ was selected as the concentration for each injection based on the observation that healthy, vitamin D replete rats had 10^-7^M 24R,25(OH)_2_D_3_ in their articular cartilage [27]. In addition, our in vitro results presented in this paper indicated that 10^-7^M 24R,25(OH)_2_D_3_ inhibited production of inflammatory cytokines in vitro.

The study was performed once using histologic assessment of tissue quality as the endpoints. Synovial fluid was collected at 28 days to evaluate effects of treatment on composition. Serum was collected at surgery and at 28 days to assess systemic effects of treatment.

#### Surgical method

20 male Sprague Dawley rats (275–300 grams) were used in the study. Surgeries were performed at Charles River Laboratories Preclinical Services in Montreal, Canada (PCS-MTL) under IACUC approval by the PCS-MTL Institutional Animal Care and Use Committee. Animals were housed in the Charles River Laboratories vivarium. ACL transection surgery was performed under isoflurane anesthesia, and all efforts were made to minimize suffering. A parapatellar skin incision was made on the medial aspect of the right knee joint and then on the medial side of the patellar tendon. The patella was then dislocated laterally to provide access to the joint space and the ACL was transected in the flexed knee. A positive anterior drawer test was performed to confirm complete transection of the ligament. The joint was then irrigated with sterile saline to avoid ancillary inflammation, and a purpose-made suture was inserted. Buprenorphine was administered as analgesic on the day of surgery (approximately 30 min before surgery and the second dose after 8-12h).

#### Intraarticular injection of 24R,25(OH)_2_D_3_

24R,25(OH)_2_D_3_ was purchased from Enzo Life Sciences (Plymouth Meeting, PA, USA) and dissolved in ethanol at a stock concentration of 10^-4^M. 40μl was then dissolved in 10 ml of sterile PBS, resulting in a final concentration of 4*10^-7^M 24R,25(OH)_2_D_3_. 24R,25(OH)_2_D_3_ or vehicle (40μl ethanol dissolved in sterile 1xPBS to a final concentration of 0.4%) was dosed immediately after the ACL surgery by irrigating the articular space with 25μl of either formulation using a micropipette. Intra-articular injections of 24R,25(OH)_2_D_3_ or vehicle (25μl) were administered every seven days until day 21 (S1 Fig). During injection, animals were maintained under general anesthesia with isoflurane. After anesthesia was achieved, the animal was restrained in a dorsal recumbent position; the hind limb articulation was shaved and wiped generously with an alcohol solution to facilitate the localization of the injection/collection site. The injection site was examined grossly by palpation of the tibia head and the patellar ligament, to identify the injection site. The needle was inserted above the tibia head and behind the patellar ligament. The injection was performed with a 29G needle by slowly releasing of the test item into the articular space.

#### Blood serum and synovial fluid collection

Blood was collected terminally on Day 28 as well as via jugular puncture on Day 1 post-surgery. Terminal bleeds were done under isoflurane anesthesia and blood (~1 mL) and was collected via abdominal aorta following which animals were euthanized by exsanguination of the abdominal aorta. All blood samples were processed for serum using standard serum separator tubes (without EDTA). On Day 28, synovial fluid lavage from both knee joints was collected following the terminal blood collection and immediately before the knee joint collection. Injection of lavage fluid (100μl 0.9% saline) was done following the intra-articular injection procedure. The limb was flexed and extended several times, and lavage fluid was drawn by re-inserting the needle at the same location.

#### Histology

Immediately after the synovial fluid collection, intact right and left knee joints were harvested and were fixed separately in neutral buffered 10% formalin. Whole knee joints were decalcified (Decal Chemical Corporation, Tallman, NY) for 16 hours on a rotating platform before being dehydrated in a series of 95% and 100% ethanol and xylene washes. Samples were embedded in paraffin. Sections (7μm thick) were stained with hematoxylin and eosin, toluidine blue, or safranin-O. Samples were imaged using a Zeiss Observer Z1 using a 20x objective. Scoring systems based on toluidine blue staining of the cartilage ECM [41] and safranin-O staining of ECM sulfated glycosaminoglycan [42] were performed. A modified Mankin scoring system was used for semi-quantitative histopathology grading for each sample [43] characterizing structural changes in the cartilage (scored 0–10), toluidine blue staining of the articular surface (scored 0–6), clone/cluster formation (scored 0–3), and loss of chondrocytes on the condyle/plateau (scored 0–6). Normal, healthy cartilage would receive a score of 0; a higher score would indicate more cartilage damage. All three scores were performed by blinded reviewers on sections taken from the same level of the joint.

#### ELISA analysis of synovial fluid and serum samples

Levels of inflammatory factors involved in OA (Bio-Plex Pro™ Multi-Plex Kit 171K1001M) and TGF-βs (1, 2, and 3) in the synovial fluid and serum were measured using a magnetic-bead-based multiplex assay (Bio-Rad, Hercules, CA, USA).

#### Statistical analysis

Data are presented as mean ± SEM of n = 10 animals per group. Data were examined by analysis of variance (ANOVA) and post hoc test using Tukey's Multiple Comparison Test. Non-parametric histomorphometry was analyzed by Mann-Whitney test, and all other data were analyzed by unpaired t-test. P<0.05 was considered to be significant.

## Results

### 24R,25(OH)_2_D_3_ blocks the effects of IL-1β on articular chondrocytes

In a preliminary set of experiments, we examined whether it would be possible to passage the rat articular chondrocytes to increase the number of cells and reduce the need for primary cultures. Phenotypic expression of chondrocyte genes was determined by real-time PCR in confluent cells at passage 1, 2, 3, and 4. Expression of mRNAs for Acan (S2A Fig), Col2a1 (S2B Fig), Sox9 (S2C Fig), Comp (S2D Fig), and Col10a1 (S2E Fig) were all markedly decreased by passage 2. In contrast, expression of mRNA for Col1a1 (S2F Fig) was increased. These results confirmed that it would be necessary to use first passage cells for our experiments, which were most similar to primary chondrocytes.

**Fig 1:**
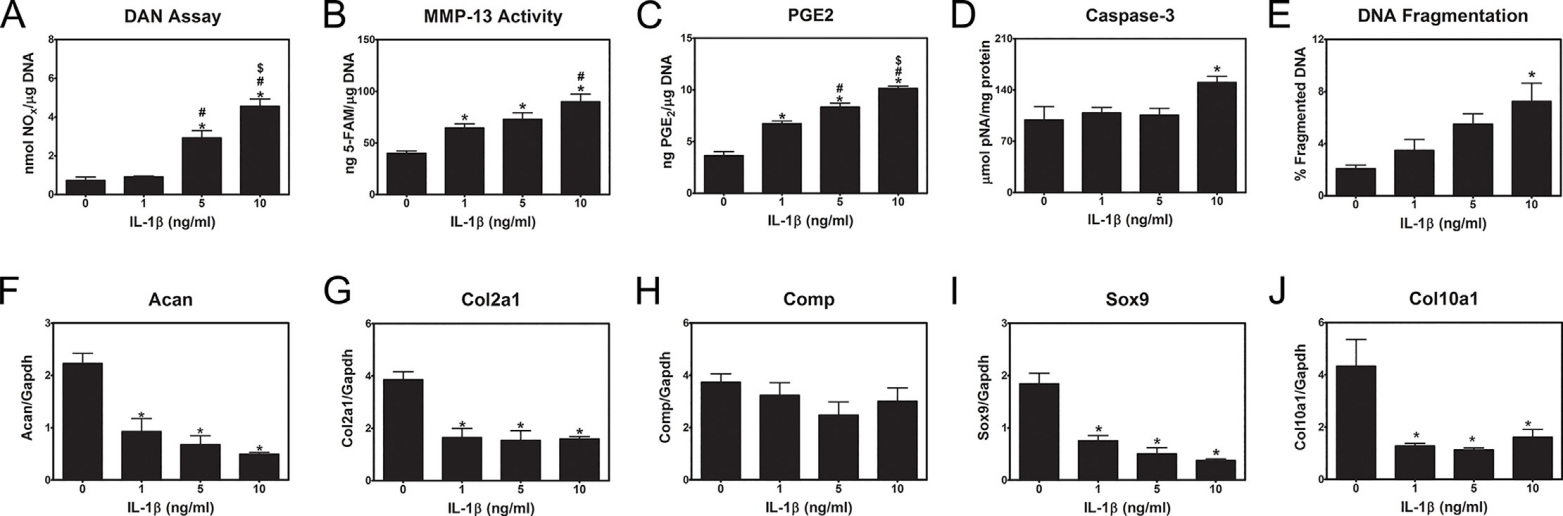
Assessment of catabolic, apoptotic, and chondrocytic mRNA levels in rat articular chondrocytes stimulated with IL-1β. First passage articular chondrocytes were treated with IL-1β for 24 hours. (**A**) NO production was measured in the conditioned media using a fluorometric assay. (**B**, **C**) MMP-13 activity and PGE2 production were measured in conditioned media using ELISA. (**D**, **E**) Apoptosis factors were measured in cell lysates using fluorometric assay and ^3^H-thymidine labeling. Amount normalized to total DNA. First passage articular chondrocytes were treated with IL-1β for 12 hours. (**F**-**J**) Expression of mRNA for chondrocyte genes was measured by real-time qPCR and normalized to Gapdh mRNA. * p<0.05 vs. control; # p<0.05 vs. 1 ng/ml IL-1β; $ p<0.05 vs. 5 ng/ml IL-1β.

The addition of IL-1β caused a dose-dependent increase in nitric oxide production (Fig 1A), MMP-13 activity (Fig 1B), and PGE2 production (Fig 1C). At the highest concentration of IL-1β, caspase-3 activity (Fig 1D) and DNA fragmentation (Fig 1E) were increased as well. Expression of chondrocyte genes including Acan (Fig 1F), Col2a1 (Fig 1G), Sox9 (Fig 1I) and Col10a1 (Fig 1J) was reduced by IL-1β at all concentrations examined. Comp (Fig 1H) mRNA were not affected by IL-1β treatment.

**Fig 2:**
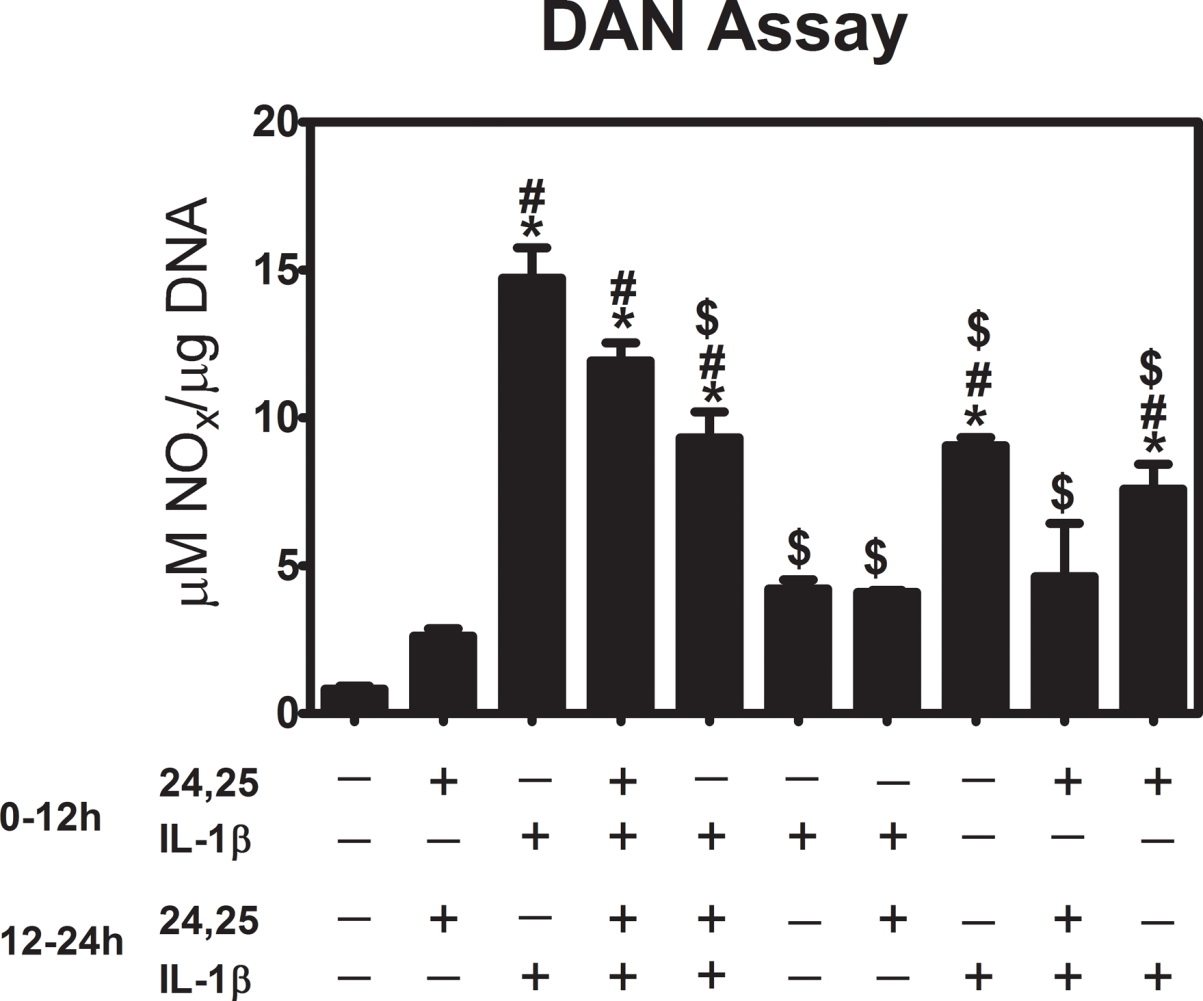
Effect of 24R,25(OH)_2_D_3_ treatment at different time points on IL-1β stimulated nitric oxide production. First passage rat articular chondrocytes were cultured with IL-1β or 24R,25(OH)_2_D_3_ for 12 hours. The media was removed, and fresh treatments of IL-1β or 24R,25(OH)_2_D_3_ were applied for another 12 hours. NO production was measured in the conditioned media, and each sample normalized to the total DNA content in the cell lysate. * p<0.05 vs. IL-1β control; # p<0.05 vs. 24R,25(OH)_2_D_3_ control; $ p<0.05 vs. 10–9 M.

In a preliminary study, we sought to establish a treatment regime for our study. To determine how 24R,25(OH)_2_D_3_ might mitigate the effects of IL-1β, we examined nitric oxide production by confluent first passage rat articular chondrocytes treated for 12 hours with either 0, 10 ng/ml IL-1β or 10^−7^ M 24R,25(OH)_2_D_3_, or the two together (Fig 2). Media were replaced at 12 hours with new media containing either vehicle alone, IL-1β, 24R,25(OH)_2_D_3_, or IL-1β plus 24R,25(OH)_2_D_3_. The DAN assay was performed at the end of the treatment. Our results show 24R,25(OH)_2_D_3_ exhibited the best inhibitory effect when it was added at the last 12 hours of the treatment (Fig 2). Therefore, all the future experiments were done by adding 24R,25(OH)_2_D_3_ for the last 12 hours.

**Fig 3:**
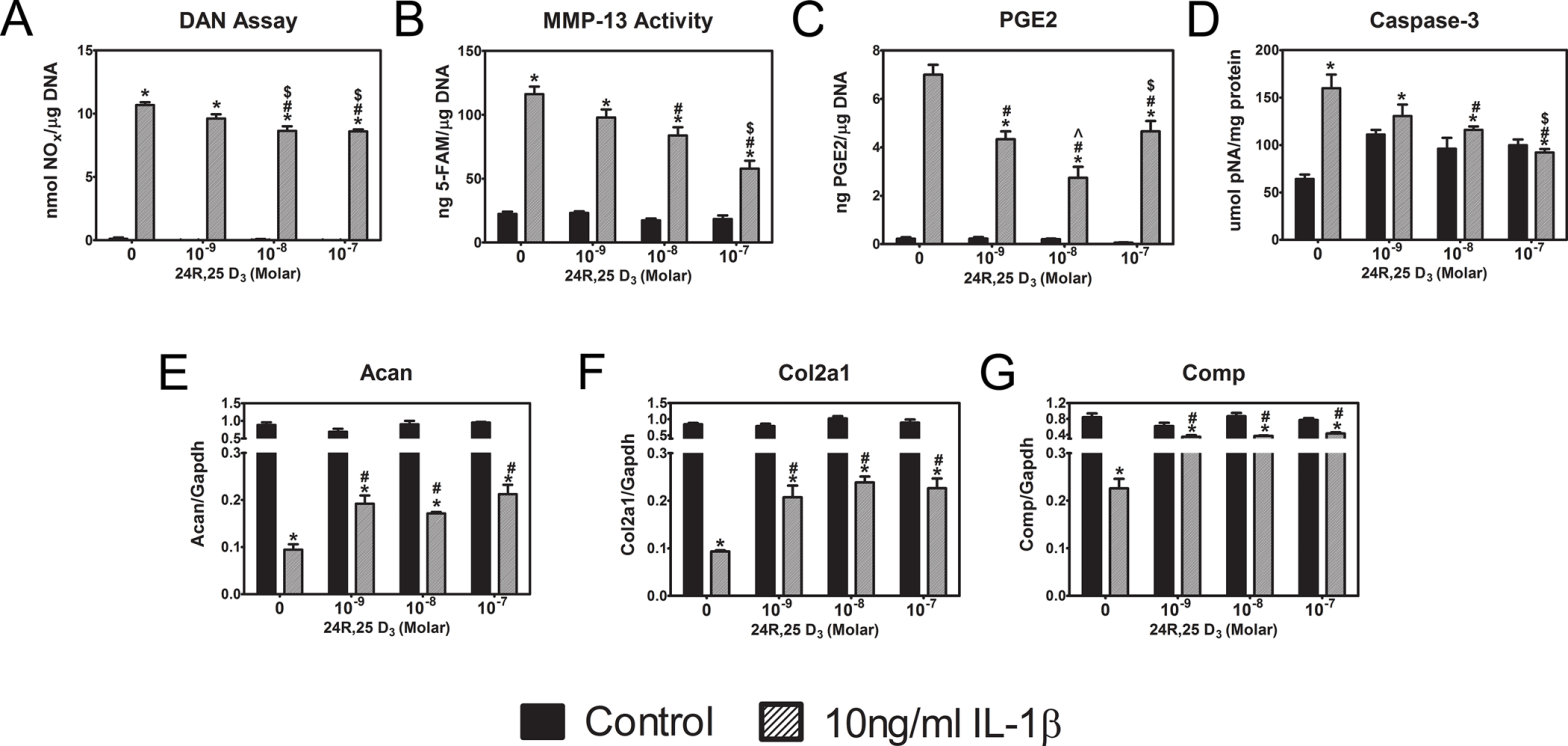
Dose-dependent effect of 24R,25(OH)_2_D_3_ treatment. First passage rat articular chondrocytes were treated with 10 ng/ml IL-1β for 12 hours. Then, the medium was exchanged and cells incubated with 10 ng/ml IL-1β containing 0, 10^−9^, 10^−8^ and 10^−7^ M 24R,25(OH)_2_D_3_. (**A**-**C**) After 24 hours, NO, MMP-13 and PGE2 levels in the conditioned media were measured and normalized to total DNA content of each sample. (**D**-**F**) After 12 hours of treatment, mRNA levels of Acan, Col2a1, and Comp were measured and normalized to Gapdh. * p<0.05 vs. IL-1β control; # p<0.05 vs. 24R,25(OH)_2_D_3_ control; $ p<0.05 vs. 10^−8^ M; ^ p<0.05 vs. 10^−7^ M.

24R,25(OH)_2_D_3_ treatment reversed inflammatory, apoptotic, and matrix decreases induced by IL-1β. By itself, 24R,25(OH)_2_D_3_ did not affect nitric oxide production (Fig 3A), MMP-13 activity (Fig 3B), PGE2 production (Fig 3C), or caspase-3 activity (Fig 3D), but it reduced the stimulatory effects of IL-1β in a dose-dependent manner. Similarly, Acan (Fig 3E), Comp (Fig 3F), and Col2a1 (Fig 3G) were not affected by treatment with 24R,25(OH)_2_D_3_, but the vitamin D metabolite partially reversed the inhibitory effect of IL-1β on expression.

The effects of 24R,25(OH)_2_D_3_ were specific. Treatment of articular chondrocytes with 1α,25(OH)_2_D_3_ did not reduce the stimulatory effect of IL-1β on MMP-13 activity (S3 Fig).

### 24R,25(OH)_2_D_3_ and TGF-β1 act in an additive manner and interact to reduce the inflammatory effects of IL-1β

**Fig 4:**
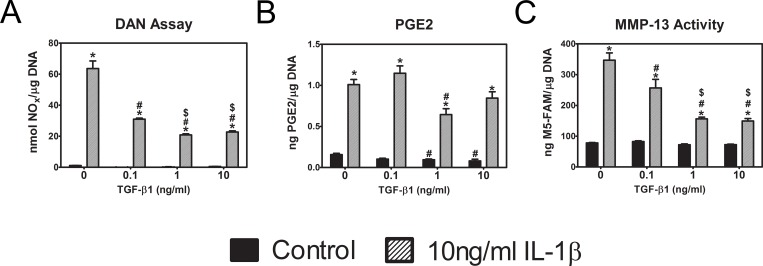
Effect of TGF- β1 signaling on IL-1 β-stimulated changes in rat articular chondrocytes. (**A**-**C**) First passage rat articular chondrocytes were treated with doses of TGF-β1 for 24 hours. Levels of NO, MMP-13 and PGE2 were measured in the conditioned media and normalized to DNA content in the cell lysate. * p<0.05 vs. TGF-β1 control; # p<0.05 vs. IL-1β control; $ p<0.05 vs. 0.1 ng/ml TGF-β1.

Previous results from our lab showed an inter-relationship between 24R,25(OH)_2_D_3_ and TGF-β1 in regulating proliferation and differentiation of costochondral cartilage. These observations suggested that the mitigating effects of 24R,25(OH)_2_D_3_ in the inflammatory response of rat articular chondrocytes might involve TGF-β1. First, we treated first passage chondrocytes for 12 hours with 0 or 10 ng/ml IL-1β. Then media were replaced with media containing 0 or 10 ng/ml IL-1β plus 0, 0.1, 1 or 10 ng/ml TGF-β1 and cultures were incubated an additional 12 hours. TGF-β1 alone had minimal effect on nitric oxide production (Fig 4A), but it reduced IL-1β-stimulated production in a dose-dependent manner. TGF-β1 had a small, but significant, effect on IL-1β-dependent increases in PGE2 production (Fig 4B). TGF-β1 caused a dose-dependent decrease in IL-1β stimulated MMP-13 activity (Fig 4C). These results were very similar to the protective effects seen after 24R,25(OH)_2_D_3_ treatment, leading us to further investigate the inter-relationship between IL-1β, TGF-β1, and 24R,25(OH)_2_D_3_.

**Fig 5:**
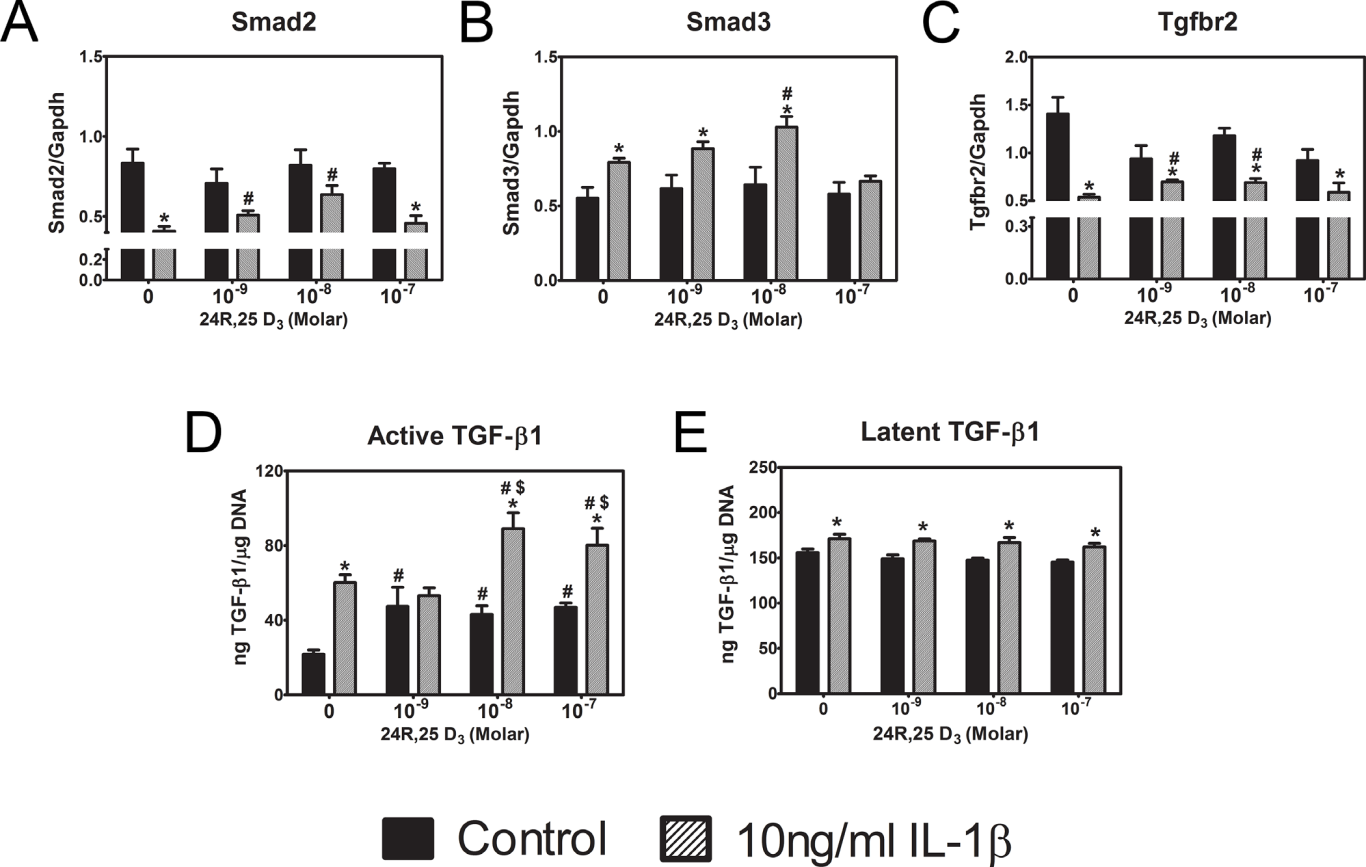
Cross talk between 24R,25(OH)_2_D_3_ and TGF-β1 signaling. First passage rat articular chondrocytes were treated with 10 ng/ml IL-1β for 12 hours. Then, the medium was exchanged and cells incubated with 10 ng/ml IL-1β containing 0, 10^−9^, 10^−8^, and 10^−7^ M 24R,25(OH)_2_D_3_. (**A-C**) After 12 hours, mRNA for TGF- β1 signaling molecules Smad 2, Smad3, and receptor Tgfbr2 were measured. * p<0.05 vs. 24R,25(OH)_2_D_3_ control; # p<0.05 vs. IL-1β control. (**D, E**) After 24 hours, active and latent TGF-β1 levels in the conditioned media were measured and normalized to DNA content in the cell lysate for each sample. * p<0.05 vs. IL-1β control; # p<0.05 vs. 24R,25(OH)_2_D_3_ control; $ p<0.05 vs. 10^−9^ M 24R,25(OH)_2_D_3_.

IL-1β has been reported to inhibit TGF-β1 signaling [44], suggesting that it might alter the availability of one or more components of the TGF-β1 signaling pathway and that 24R,25(OH)_2_D_3_ may act to reverse this. IL-1β reduced Smad2 expression, and this was partially reversed by 10^−8^ M 24R,25(OH)_2_D_3_ (Fig 5A). IL-1β caused an increase in Smad3 expression, and this was enhanced by the inclusion of 10^−8^ M 24R,25(OH)_2_D_3_ but was returned to control levels at 10^−7^ M (Fig 5B). IL-1β caused a marked reduction in Tgfbr2 expression, and this was partially reversed by 24R,25(OH)_2_D_3_ (Fig 5C). Importantly, IL-1β and 24R,25(OH)_2_D_3_ both increased active TGF-β1 when cells were treated with either factor alone (Fig 5D). However, when cells were treated with both factors in combination, the effects were additive. In contrast, latent TGF-β1 levels were not affected by either factor (Fig 5E).

**Fig 6:**
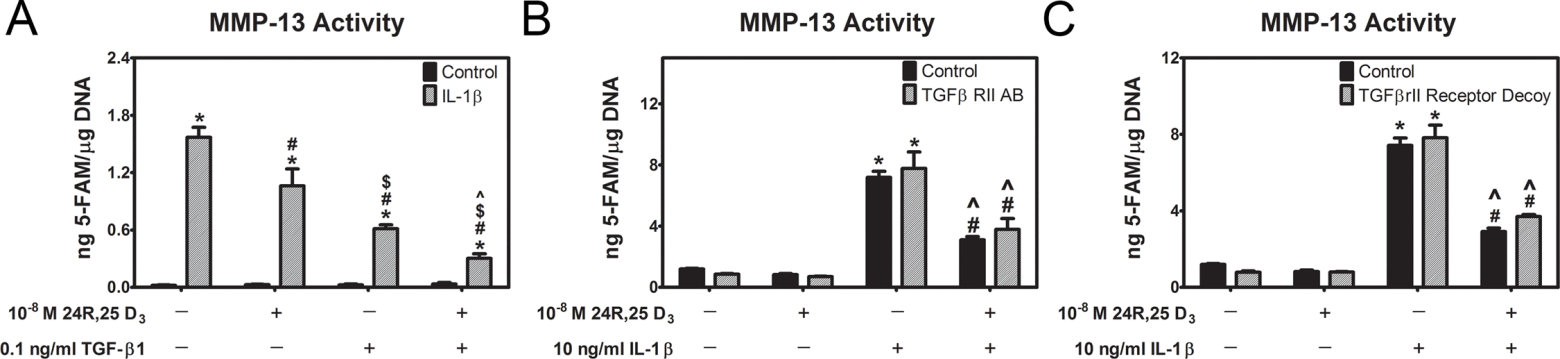
Effect of 24R,25(OH)_2_D_3_ is not mediated through TGF-β1 signaling. (**A**) Effect of 0.1 ng/ml TGF-β1 with 10^−8^ M 24R,25(OH)_2_D_3_ co-treatment on IL-1β induced MMP-13 activity. Levels normalized to total DNA. * p<0.05 vs. IL-1β control; # p<0.05 vs. 24R,25(OH)_2_D_3_ control; $ p<0.05 vs.10^-8^ M 24R,25(OH)_2_D_3_; ^ p<0.05 vs. 0.1 ng/ml TGF-β1. (**B**, **C**) Effect of blocking TGF-β1 signaling using either an antibody against TGF-β1rII or a decoy receptor on MMP-13 activity in cells treated with IL-1β and 24R,25(OH)_2_D_3_. Levels normalized to total DNA in cell lysates of each sample. * p<0.05 vs. IL-1β control; # p<0.05 vs. 24R,25(OH)_2_D_3_ control; ^ p<0.05 vs. 10 ng/ml IL-1β.

To further clarify the interaction between 24R,25(OH)_2_D_3_ and TGF-β1 in mitigating the response to IL-1β, we set up a matrix study design using MMP-13 activity as the outcome measure. Confluent first passage chondrocytes were cultured for 12 hours with 0 or 10 ng/ml IL-1β. At that time, media were replaced with media containing IL-1β plus either 24R,25(OH)_2_D_3_ or TGF-β1. Borth 24R,25(OH)_2_D_3_ and TGF-β1 reduced IL-1β’s effect, and treatment with the two factors together caused an additive reduction in MMP-13 activity (Fig 6A). In a second study, cultures were treated with 0 or 10 ng/ml IL-1β for 12 hours and the media were then replaced with media containing either IL-1β or 24R,25(OH)_2_D_3_. A blocking antibody to the TGF-β1 type II receptor was added to one-half of the cultures (Fig 6B). By itself, 24R,25(OH)_2_D_3_ had no effect on MMP-13 activity; however, when the cultures were treated with IL-1β, activity was stimulated, but it was not affected by the inclusion of the antibody. A similar result was found when the cultures were treated with a receptor inhibitor rather than the blocking antibody (Fig 6C). These results demonstrated that the protective effects of 24R,25(OH)_2_D_3_ were not mediated through TGF-β1.

### 24R,25(OH)_2_D_3_ prevents OA changes due to ACL transection in immunocompetent rats

**Fig 7:**
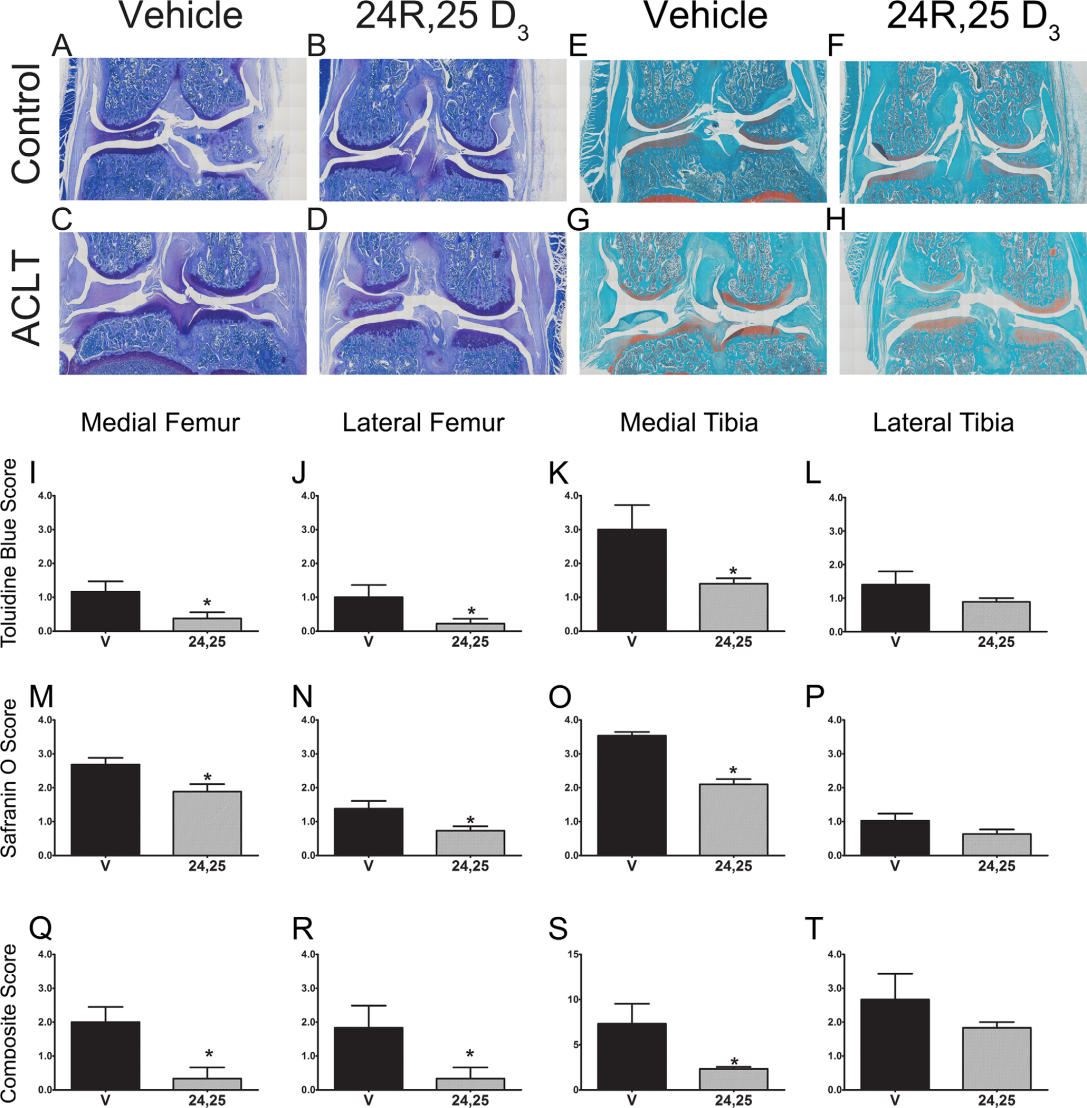
Histopathological staining and scoring of rat articular joints. Male Sprague-Dawley rats underwent ACLT and allowed to recover for 28 days. (**A**-**D**) Representative images of toluidine blue staining of vehicle or 24R,25(OH)_2_D_3_ treated rat knees after ACLT as well as contralateral control knees. (**E-H**) Representative images of safranin O staining of vehicle or 24R,25(OH)_2_D_3_ treated rat knees after ACLT as well as contralateral control knees. (**I**-**L**) Scores for toluidine blue staining for four quadrants of rat knees that were treated with either vehicle or 24R,25(OH)_2_D_3_ from histological sections taken at the same level of the joint. (**M**-**P**) Scores for safranin-O staining for four quadrants of rats knee that were treated with either vehicle or 24R,25(OH)_2_D_3_ from histological sections taken at the same level of the joint. (**Q-T**) Total composite scores of rat knees that were treated with either vehicle or 24R,25(OH)_2_D_3_ using a modified Mankin system from histological sections taken at the same level of the joint. * p<0.05 vs. control.

We next wanted to examine whether 24R,25(OH)_2_D_3_ was chondroprotective in an in vivo model of osteoarthritis whereby the ACL is severed, destabilizing the knee and leading to joint inflammation and damage to the articular cartilage. At 28 days after ACL transection, OA changes were evident in the knees injected with vehicle only (Fig 7C). They appeared as roughened patches on the articular surface of the medial and lateral femur and medial tibia, but not on the lateral tibia or any of the cartilage surfaces of the contralateral knees (Fig 7A and 7B). In contrast, surface damage was significantly reduced when the operated knees were treated with 24R,25(OH)_2_D_3_. Histology of the knees confirmed this. Whereas control knees exhibited full thickness cartilage on all articulating surfaces (Fig 7A), cartilage was markedly reduced on the medial and lateral femur and absent from the medial tibia when transected joints were injected with PBS containing only vehicle (Fig 7C). However, when the transected joints were treated with 24R,25(OH)_2_D_3_, cartilage thickness was reduced compared to control knees, but it was still present on all surfaces (Fig 7D). Treatment with 24R,25(OH)_2_D_3_ markedly reduced damage to the medial and lateral femur by over 60% and to the medial tibia by 50% but did not alter the toluidine positive tissue on the lateral tibia (Fig 7I–7L, respectively).

Sections of the knee stained with safranin-O, which stains sulfated glycosaminoglycans, told a similar story. Contralateral control knees had full thickness cartilage on all articulating surfaces (Fig 7E). The medial femur and medial tibia of knees treated with the vehicle after ACL transfection exhibited markedly reduced safranin O positive stain (Fig 7G). While the lateral femur and tibia were less affected, the stain was still reduced compared to the contralateral controls. Safranin-O staining was reduced on all joint surfaces compared to the contralateral controls in knees treated with 24R,25(OH)_2_D_3_ (Fig 7H), but the contours of the articular cartilage surfaces were smooth and intact (Fig 7F). Quantitative scoring supported these morphological observations. The safranin-O scores for the medial femur, lateral femur, and medial tibia were all lower in the 24R,25(OH)_2_D_3_-treated joints, whereas no differences were detected in the lateral tibia (Fig 7M–7P, respectively).

When all measurements of cartilage health were compiled as a composite score using a modification of the Mankin system [45,46], 24R,25(OH)_2_D_3_ reduced damage to the medial femur by more than 80% (Fig 7Q), to the medial tibia by 75% (Fig 7R), and to the lateral femur by 90% (Fig 7S), but did not affect cartilage health in the lateral tibia (Fig 7T).

### Intra-articular treatment with 24R,25(OH)_2_D_3_ modulates the composition of synovial fluid

**Fig 8:**
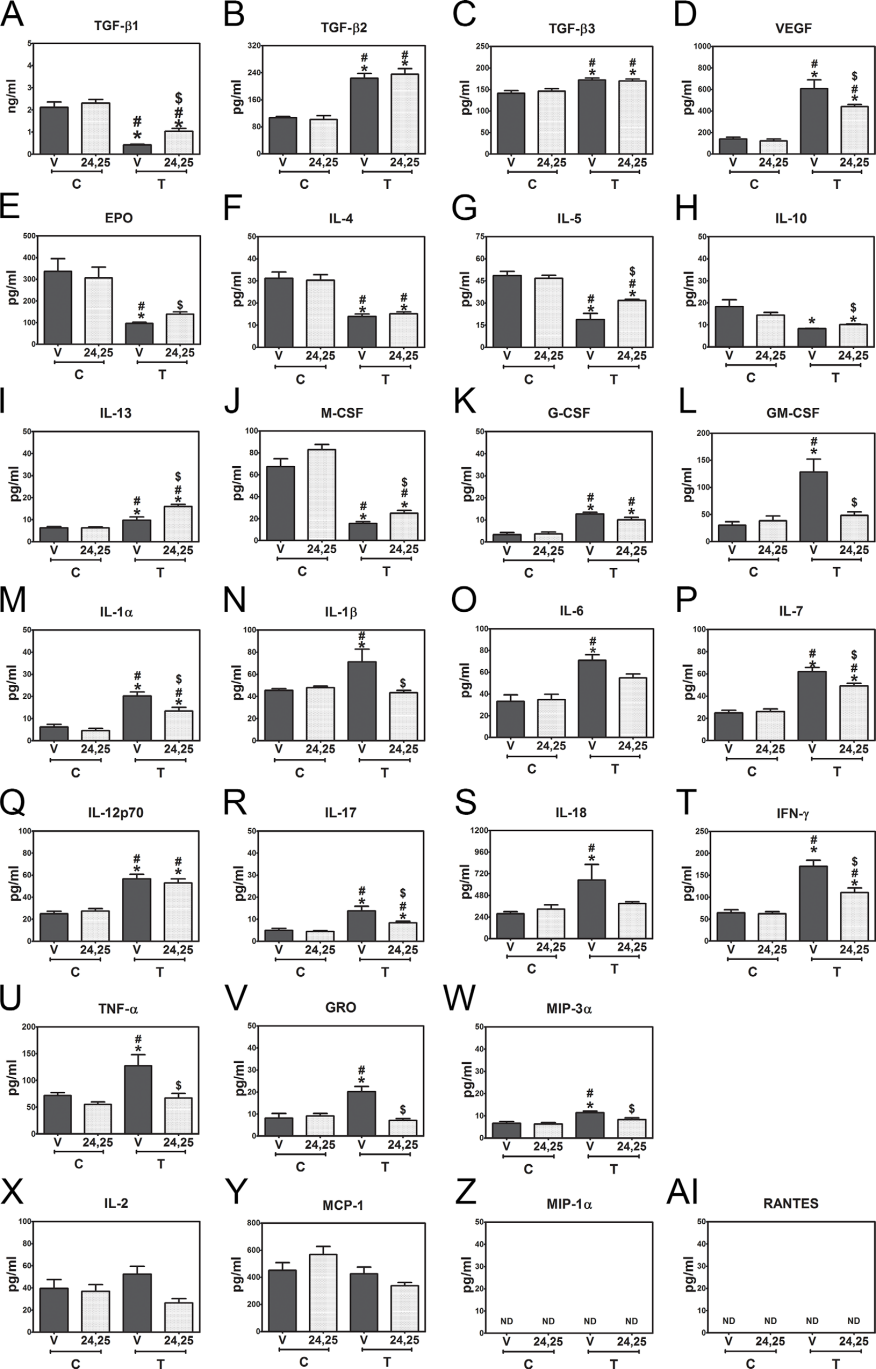
Synovial fluid profile of cytokines, chemokines and growth factors. Male Sprague-Dawley rats underwent ACLT and allowed to recover for 28 days. Factors were measured at the end of the study using a magnetic-bead-based multiplex ELISA. (**A**-**D**) Growth factors involved in cartilage remolding during osteoarthritis. (**E**-**J**) Anti-inflammatory factors involved in osteoarthritis. (**K-W**) Pro-inflammatory factors found in osteoarthritis. (**X-AI)** Cytokines and inflammatory factors. T represents the ACLT knee with either vehicle or 24R,25(OH)_2_D_3_. C represents contralateral control knee for either vehicle or 24R,25(OH)_2_D_3_ group. * p<0.05 vs. vehicle contralateral control knee; # p<0.05 vs. contralateral 24R,25(OH)_2_D_3_ knee; $ p<0.05 vs. vehicle treated knee.

To better understand the changes in tissue response, we examined the effects of the injection protocol on the composition of a broad range of factors associated with inflammation and arthritis in the synovial fluid (Fig 8A–8AI). Overall, the intra-articular injections of PBS containing either ethanol vehicle alone or 24R,25(OH)_2_D_3_ in the operated knee did not affect the composition of the synovial fluid of the non-injected contralateral knees at 28 days post-surgery. However, the composition of the synovial fluid in the operated knees was impacted by the OA changes induced by ACL transection and was differentially affected by injection with 24R,25(OH)_2_D_3_. For most of the factors assayed, 24R,25(OH)_2_D_3_ mitigated the effect of ACL transection and treatment with PBS+vehicle, either by reducing its stimulatory effect or by increasing its inhibitory effect compared to levels in the contralateral knee.

We categorized the proteins based on the potential of each factor to contribute to reduced inflammation and tissue healing versus increased inflammation and tissue degeneration. TGF-β1 was markedly reduced in synovial fluid from operated knees treated with PBS+vehicle compared to the contralateral control (Fig 8A). In contrast, TGF-β2 (Fig 8B) and TGF-β3 (Fig 8C) were increased. 24R,25(OH)_2_D_3_ partially blocked the reduction in TGF-β1, but it had no effect on the increase in TGF-β2 or TGF-β3. VEGF was increased more than three-fold in the operated knees treated with PBS+vehicle compared to the contralateral knees (Fig 8D); 24R,25(OH)_2_D_3_ caused a significant reduction in VEGF content. Effects on erythropoietin (EPO, Fig 8E) were reversed from those on VEGF. 24R,25(OH)_2_D_3_ had no effect on synovial fluid IL-4 in the operated knees (Fig 8F), but it did mitigate the effects of ACL transection plus treatment with PBS+vehicle on IL-5 (Fig 8G) and IL-10 (Fig 8H). IL-13, which has been shown to protect synoviocytes from apoptosis in rheumatoid arthritis [47], was upregulated by treatment with vehicle alone and further increased by 24R,25(OH)_2_D_3_ (Fig 8I), which demonstrates the complexity of events in the knee joint. Treatment with vehicle alone reduced macrophage colony-stimulating factor (M-CSF), and 24R,25(OH)_2_D_3_ partially restored this (Fig 8J).

Factors that are associated with tissue degradation or inflammation were also differentially present in the synovial fluid. In all cases, synovial fluid from operated knees treated with PBS+vehicle had higher levels than the contralateral knee (Fig 8K–8W). Treatment with 24R,25(OH)_2_D_3_ partially reduced the content of granulocyte-colony stimulating factor (G-CSF, Fig 8K) and completely blocked the effect of PBS+vehicle on granulocyte macrophage colony-stimulating factor (GM-CSF, Fig 8L). The effect of 24R,25(OH)_2_D_3_ on levels of interleukins in synovial fluid varied: IL-1α was partially reduced (Fig 8M); IL-1β was reduced to levels seen in the contralateral knees(Fig 8N); IL-4 was lower, but this difference was not statistically significant (Fig 8O); IL-7 was lower (Fig 8P); IL-12p70 was unaffected (Fig 8Q); IL-17 was reduced (Fig 8R); and IL-18 was reduced to levels seen in the contralateral knees (Fig 8S). Interferon-gamma (IFN-γ) was partially reduced by 24R,25(OH)_2_D_3_ (Fig 8T), but tumor necrosis factor-alpha (TNF-α, Fig 8U), growth-related oncogene (GRO, Fig 8V) and macrophage inflammatory protein-3 alpha (MIP-3α, Fig 8W) were all reduced to levels observed in the contralateral knees. The levels of IL-2 (Fig 8X) and MCP-1 (Fig 8Y) in the synovial fluid were unaffected by ACL transection or by injection with PBS+vehicle or 24R,25(OH)_2_D_3_. Neither MIP-1α (Fig 8Z) nor regulated on activation, normal T cell expressed and secreted (RANTES, Fig 8AI) was detected in any synovial fluid samples.

### Intra-articular injection of 24R,25(OH)_2_D_3_ increases anti-inflammatory cytokines and reduces inflammatory cytokines in the serum

**Fig 9:**
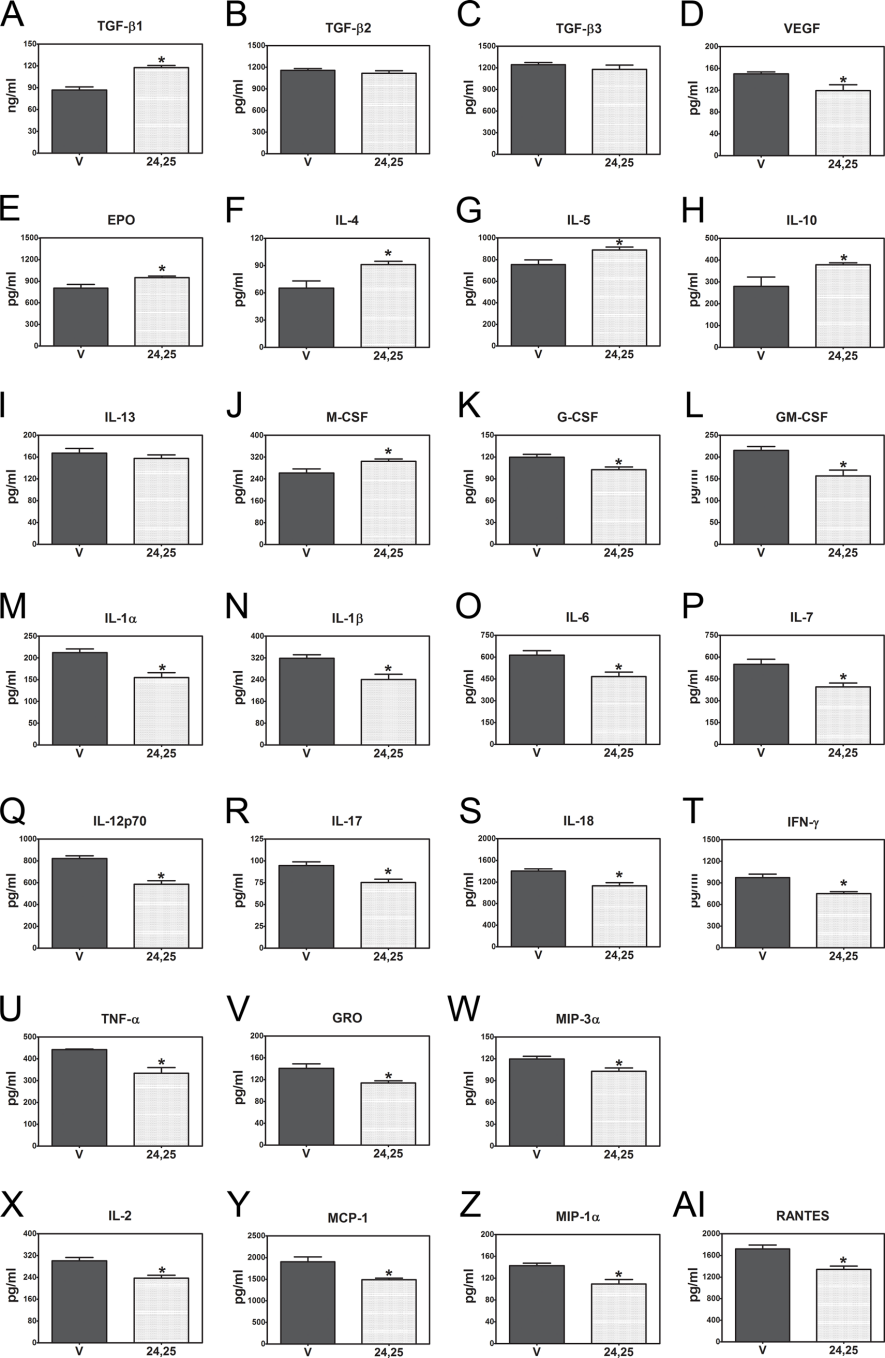
Serum levels of cytokines, chemokines, and growth factors on postoperative day 28. Male Sprague-Dawley rats underwent ACLT and allowed to recover for 28 days. Factors were measured in the serum of rats that were treated with either vehicle or 24R,25(OH)_2_D_3_ at day 28 immediately before euthanasia. (**A**-**D**) Growth factors involved in cartilage remodeling during osteoarthritis. (**E**-**J**) Anti-inflammatory factors involved in osteoarthritis. (**K**-**W**) Pro-inflammatory factors found in osteoarthritic knees. (**X-AI**) Cytokines and inflammatory factors. * p<0.05 vs. vehicle control group.

With the exception of serum TGF-β1 (S4A Fig) and IL-10 (S4H Fig), which were increased by day 1 following injection of 24R,25(OH)_2_D_3_, no other changes in serum factors were evident on day 1 as a function of intra-articular injection with either PBS+vehicle or 24R,25(OH)_2_D_3_ (S4B–S4G and S4I–S4W Fig). At 28 days post-surgery, TGF-β1 was elevated in rats treated with 24R,25(OH)_2_D_3_ at levels comparable to those seen on day 1 (Fig 9A). However, neither TGF-β2 (Fig 9B) nor TGF-β3 (Fig 9C) was affected. 24R,25(OH)_2_D_3_ treatment of the operated knees caused a decrease in serum VEGF (Fig 9D) and an increase in serum EPO (Fig 9E). IL-4 (Fig 9F), IL-5 (Fig 9G), and IL-10 (Fig 9H) were increased; IL-13 was unaffected (Fig 9I), and M-CSF (Fig 9J) was increased in animals treated with 24R,25(OH)_2_D_3_. Importantly, treatment of the ACL-transected knee with 24R,25(OH)_2_D_3_ resulted in a decrease in all of the inflammatory cytokines assayed in comparison to knees injected with PBS+vehicle alone (Fig 9K–9W). IL-2 (S4X Fig), MIP-1α (S4Z Fig), and RANTES (S4AI Fig) were all detected in serum one day post-operatively, but their levels were not affected by 24R,25(OH)_2_D_3_. MCP-1 (S4Y Fig) was downregulated on day 1 in animals receiving by 24R,25(OH)_2_D_3_. However, on day 28, the levels of all four factors were reduced in serum from animals treated with the vitamin D metabolite (Fig 9X–9AI).

## Discussion

In the present study, we used articular chondrocytes treated with IL-1β as a surrogate for inflammation-induced cartilage damage [48,49]. The results showed that 24R,25(OH)_2_D_3_ reduced the stimulatory effects of IL-1β on the production of reactive oxygen species, activity of MMP-13, production of PGE2, and caspase-3 activity. The effects of 24R,25(OH)_2_D_3_ were specific; 1α,25(OH)_2_D_3_, which is also produced in articular cartilage [27], did not reduce the stimulatory effect of IL-1β on MMP-13. 24R,25(OH)_2_D_3_ also reversed the inhibitory effects of IL-1β on cartilage matrix synthesis. We have shown that 24R,25(OH)_2_D_3_ inhibits apoptosis induced by a number of apoptogens [25,26,50] via PLD-dependent signaling [51,52], and this study further supports its chondroprotective effects.

Our results show that acute intra-articular treatment with 24R,25(OH)_2_D_3_ can prevent the development of OA following transection of the ACL in Sprague Dawley rats. Our data demonstrate that OA damage following ACL transection in rats is associated with a number of changes in synovial fluid composition. Factors that elicit inflammation, including Il-1β were increased as was enzyme activity that these factors stimulate. Anti-inflammatory factors were reduced. Treatment with 24R,25(OH)_2_D_3_ has the opposite effect. Pro-inflammatory factors were reduced, anti-inflammatory factors were increased, and activity of the matrix processing enzymes was reduced.

To test the effects of 24R,25(OH)_2_D_3_ on osteoarthritis *in vivo*, we chose the ACL transection model for its clinical relevance and applicability to translational research. Others have shown that the changes seen in the cartilage in this model follow the changes observed in human cartilage after ACL rupture [53,54]. Our results indicate that acute intra-articular treatment with 24R,25(OH)_2_D_3_ can prevent the development of OA following transection of the ACL in Sprague Dawley rats. Previous studies using this model have tested technologies to reverse OA damage [55–58]. Our results suggest that a series of weekly injections of 24R,25(OH)_2_D_3_ immediately following ACL trauma and during the early healing period reduces inflammatory markers and may prevent OA changes in the articular cartilage. Destabilization of the knee resulted in the loss of cartilage matrix sulfated glycosaminoglycans and fibrillation of the surface. This outcome was markedly reduced in knees that were treated with the vitamin D metabolite.

24R,25(OH)_2_D_3_ is normally present in the blood as a consequence of hydroxylation of 25(OH)D_3_ in the kidney [59]. Recently, we demonstrated that 24,25(OH)_2_D_3_ is present in synovial fluid aspirated from human knees removed during total knee arthroplasty [60]. It is not clear whether this represents diffusion from the vasculature or is due to local production by knee tissues. Chondrocytes possess the ability to hydroxylate 25(OH)D_3_ to both 1,25(OH)_2_D_3_ and 24,25(OH)_2_D_3_, and this is regulated by hormones and growth factors [28,61], suggesting the content of vitamin D metabolites in the knee is essential for cartilage health. The observation that low serum 25(OH)D_3_ is correlated with OA [62] supports this.

TGF-β1 elicited outcomes similar to 25R,25(OH)_2_D_3_ using the same cell culture model and was additive with 24R,25(OH)_2_D_3_ in blocking responses to IL-1β. While this shows that each factor acts via an independent mechanism, our results also support the involvement of an inter-dependent mechanism. 24R,25(OH)_2_D_3_ blocked the reduction in Smad2 and increase in Smad3 caused by IL-1β, and reduced the inhibition of the TGF-β type II receptor. Whereas Smad2 mediates TGF-β1 signaling controlling proliferation, apoptosis, and differentiation, Smad3 induces the repression of target genes, particularly c-myc [63]. IL-1β caused a small but significant increase in latent TGF-β1 that was unaffected by 24R,25(OH)_2_D_3_. However, 24R,25(OH)_2_D_3_ markedly increased levels of active TGF-β1 even in IL-1β treated cells, suggesting that the increase in TGF-β1 noted in vivo was specifically due to direct effects of 24R,25(OH)_2_D_3_ on activation and release of the growth factor. TGF-β1 can then act back on the cells to stimulate ECM production [64–66] and to reduce inflammation [67–69].

Intra-articular injection of 24R,25(OH)_2_D_3_ caused a rapid increase in serum TGF-β1 within 24 hours, and this remained elevated to the same extent on day 28. TGF-β1 was first identified as a chondrogenic factor present in guanidine-HCl extracts from cartilage [70] and has been used to induce chondrogenesis in cultures of mesenchymal stem cells [71–73]. It is present in cartilage ECM in the latent form [70], and its storage in the matrix via latent TGF-β binding protein is regulated by 24R,25(OH)_2_D_3_ [30]. We found that TGF-β1 present in synovial fluid was markedly reduced in ACL-transected knees injected with PBS in comparison to contralateral control knees. In contrast, levels were higher in synovial fluid treated with 24R,25(OH)_2_D_3_. Whether this was due to the greater synthesis of TGF-β1 or to the regulation of TGF-β1 storage via latent TGF-β binding protein or to altered activation of latent TGF-β1 is unknown. It is evident, however, that the effects are specific to 24R,25(OH)_2_D_3_ and TGF-β1, and support the changes seen in chondrocyte phenotype in our cell culture model. TGF-β2 and TGF-β3 were both elevated in synovial fluid from ACL-transected joints compared to contralateral knees, but 24R,25(OH)_2_D_3_ had no effect. Also, serum levels of both TGF-β2 and TGF-β3 were unaffected. It is also interesting to note that after only four injections of 24R,25(OH)_2_D_3_ into the joint, we were able to observe such changes in the serum.

Our results indicate that many factors are regulated by 24R,25(OH)_2_D_3_ and are likely to contribute to the overall anabolic effect of the hormone. While we have focused on IL-1β and TGF-β1, it is clear that there is a complex milieu generated in the joint by 24R,25(OH)_2_D_3_. Vitamin D metabolism also complicates the story. 25(OH)D_3_ is hydroxylated on the 1-carbon by cytochrome p450 27B1 (Cyp27B1). In chondrocytes, this is regulated by TGF-β1 [61]. Alternatively, it can be hydroxylated on the 24-carbon by Cyp24A1, and this is regulated by 1α,25(OH)_2_D_3_ [74]. Thus, some of the effects attributed to 24R,25(OH)_2_D_3_ may also reflect actions of other vitamin D metabolites. Future studies must also examine osteoarthritis in the Cyp24a1-/- mouse, which lacks 24,25(OH)_2_D_3_, to definitively demonstrate the role of 24,25(OH)_2_D_3_ in osteoarthritis progression.

Our results suggest that 24R,25(OH)_2_D_3_ reduced activity of enzymes associated with tissue damage, reduced apoptosis, or reduced production of inflammatory mediators as well as to stimulation of matrix synthesis in articular cartilage in osteoarthritic conditions in vitro and in vivo. Acute intra-articular injection of 24R,25(OH)_2_D_3_ may prevent the development of articular cartilage damage after trauma, and reduce or prevent early onset osteoarthritis.

## Supporting Information

S1 Fig Animal study design. Schematic showing injection timeline and study design of in vivo 24R,25(OH)_2_D_3_ injection in rat knees.
(TIF)

S2 Fig mRNA levels of chondrocyte phenotype genes in rat articular chondrocytes in cells cultured to different passages. Articular chondrocytes were isolated from rats and cultured. At passages 1–4, RNA was extracted and the chondrocyte phenotype characterized by mRNA levels of Acan (A), Col2a2 (B), Sox9 (C), Comp (D), Col10a1 (E), and Col1a1 (F). *p<0.05 vs. P1.
(TIF)

S3 Fig Effect of 24R,25(OH)_2_D_3_ is specific to the vitamin D metabolite. First passage rat articular chondrocytes were treated with 10 ng/ml IL-1β for 12 hours. Then, the medium was exchanged and cells incubated with 10 ng/ml IL-1β containing either full medium, 10–7 M 24R,25(OH)_2_D_3_, or 10–8 M 1α,25(OH)2D3. After 24 hours, MMP activity was measured and normalized to DNA content in the cell lysate for each sample. *p<0.05 vs. untreated cells; #p<0.05 vs. no IL-1β treatment; $p<0.05 vs. IL-1β treatment; ^p<0.05 vs. 24R,25(OH)_2_D_3_ treatment.
(TIF)

S4 Fig Serum levels of cytokines, chemokines, and growth factors one day following ACLT. (A-D) Growth factors involved in cartilage remolding during osteoarthritis. (E-J) Anti-inflammatory factors involved in osteoarthritis. (K-W) Pro-inflammatory factors found in osteoarthritic knees. (X-AI) Cytokines and inflammatory factors. * p<0.05 vs. vehicle control group.
(TIF)
