# Supplementary figures and images for: 24R,25-Dihydroxyvitamin D3 Protects against Articular Cartilage Damage following Anterior Cruciate Ligament Transection in Male Rats
Source: PLoS One. 2016 Aug 30;11(8):e0161782. doi: 10.1371/journal.pone.0161782 (PMC5019362; doi:10.1371/journal.pone.0161782)

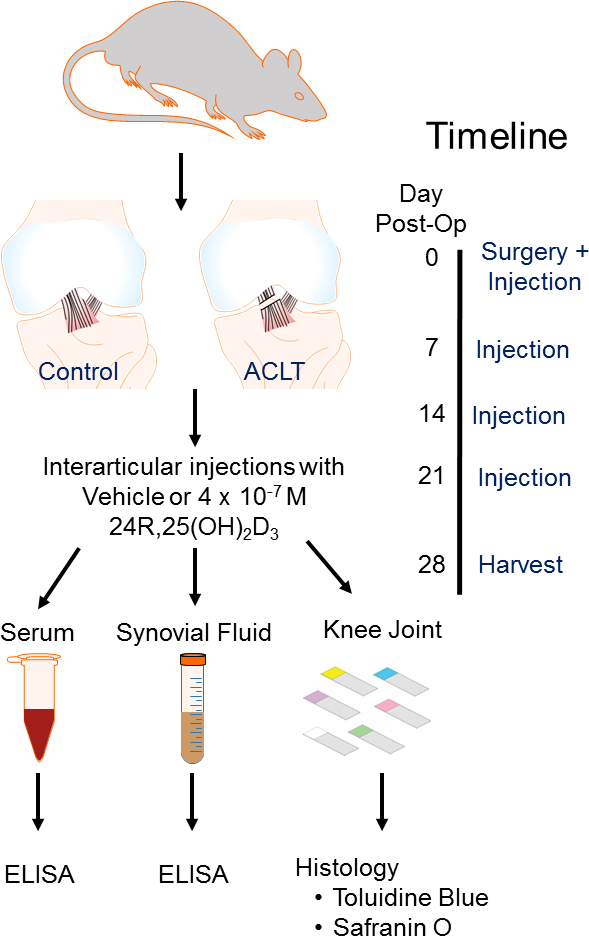

Supplement: S1 Fig — Schematic showing injection timeline and study design of in vivo 24R,25(OH)2D3 injection in rat knees. (TIF) [file pone.0161782.s001.tif]

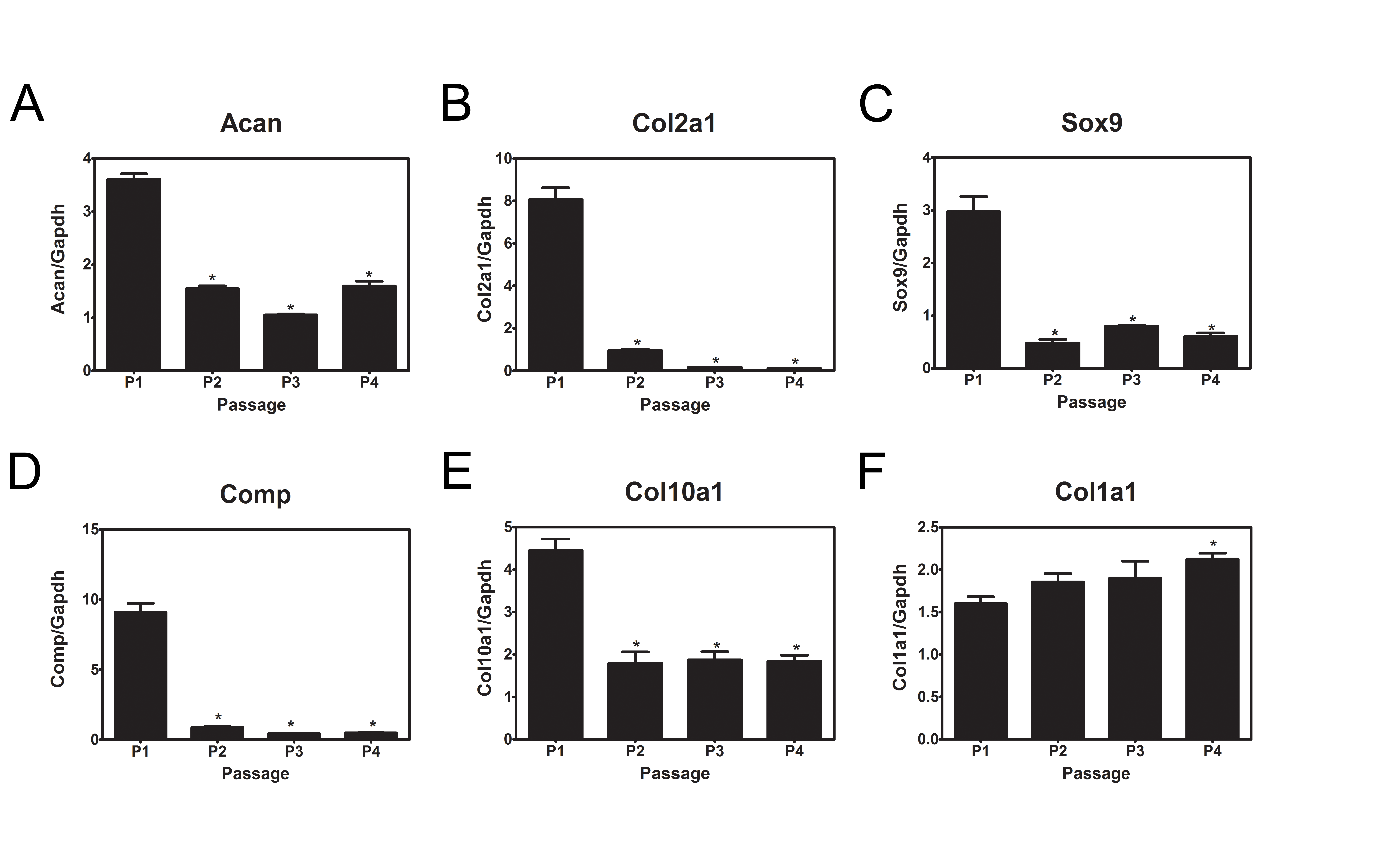

Supplement: S2 Fig — Articular chondrocytes were isolated from rats and cultured. At passages 1–4, RNA was extracted and the chondrocyte phenotype characterized by mRNA levels of Acan (A), Col2a2 (B), Sox9 (C), Comp (D), Col10a1 (E), and Col1a1 (F). *p<0.05 vs. P1. (TIF) [file pone.0161782.s002.tif]

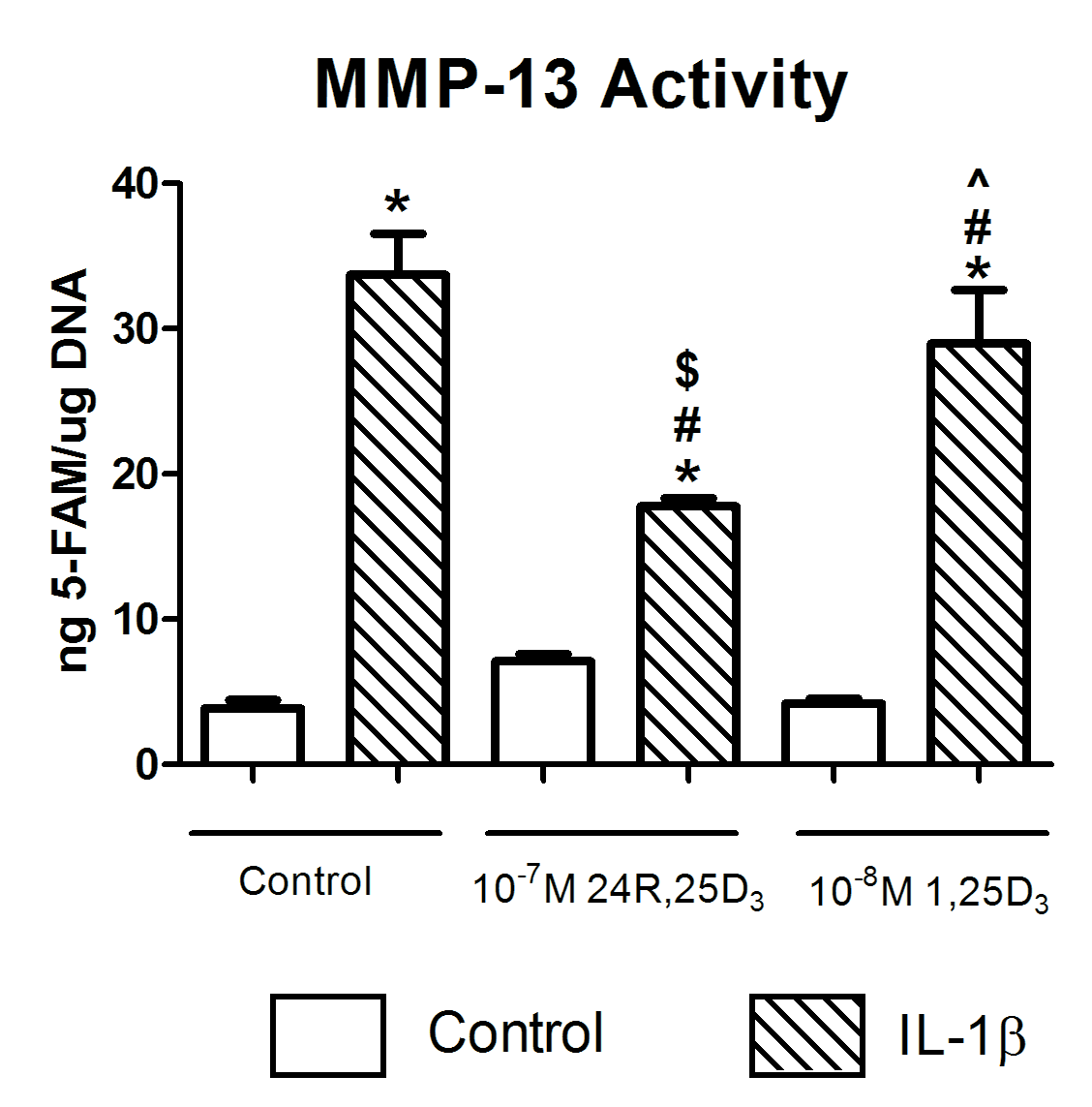

Supplement: S3 Fig — First passage rat articular chondrocytes were treated with 10 ng/ml IL-1β for 12 hours. Then, the medium was exchanged and cells incubated with 10 ng/ml IL-1β containing either full medium, 10–7 M 24R,25(OH)2D3, or 10–8 M 1α,25(OH)2D3. After 24 hours, MMP activity was measured and normalized to DNA content in the cell lysate for each sample. *p<0.05 vs. untreated cells; #p<0.05 vs. no IL-1β treatment; $p<0.05 vs. IL-1β treatment; ^p<0.05 vs. 24R,25(OH)2D3 treatment. (TIF) [file pone.0161782.s003.tif]

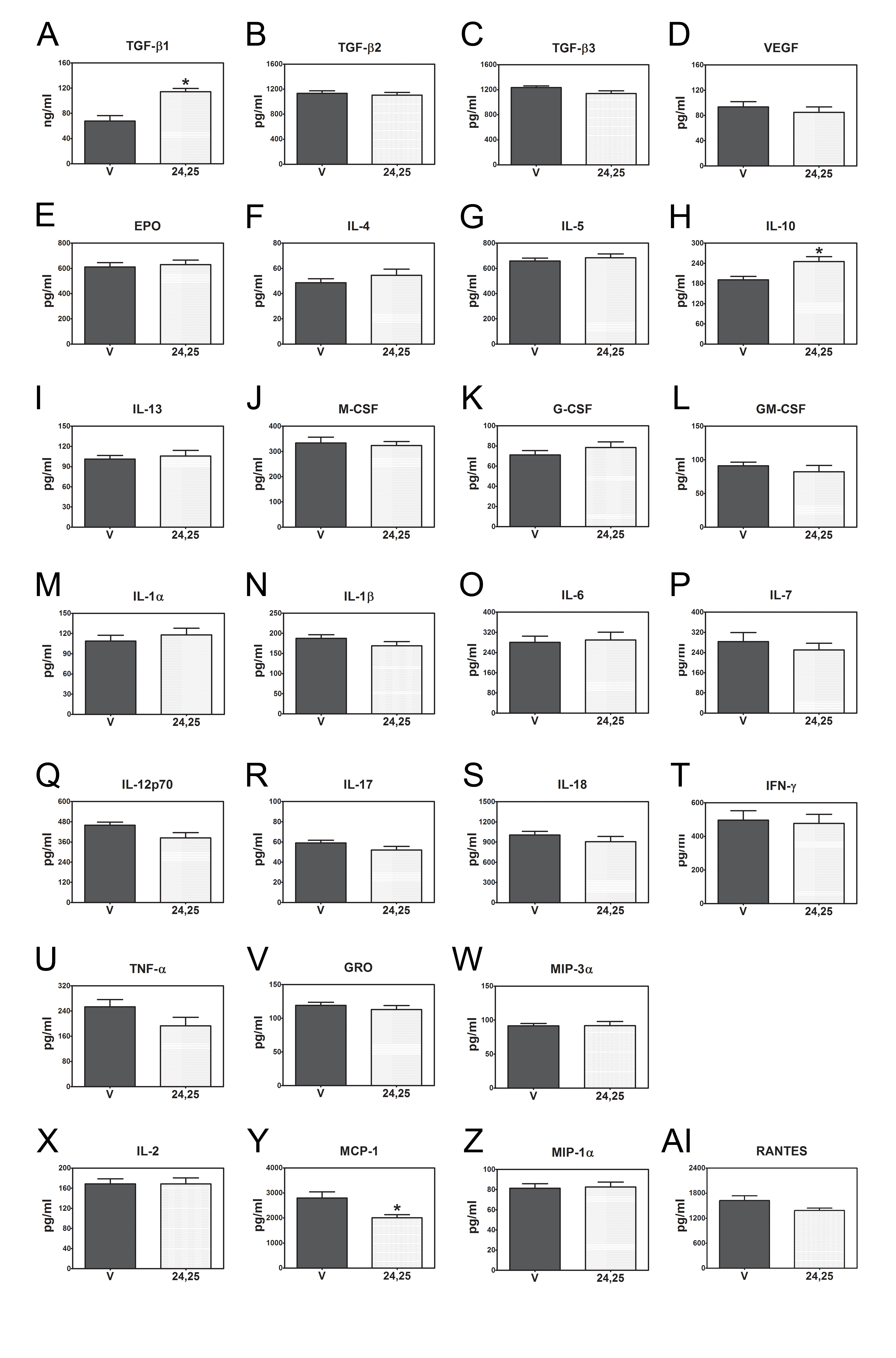

Supplement: S4 Fig — (A-D) Growth factors involved in cartilage remolding during osteoarthritis. (E-J) Anti-inflammatory factors involved in osteoarthritis. (K-W) Pro-inflammatory factors found in osteoarthritic knees. (X-AI) Cytokines and inflammatory factors. * p<0.05 vs. vehicle control group. (TIF) [file pone.0161782.s004.tif]
